# Blood-based biomarkers for Alzheimer’s disease diagnosis: a joint position paper from the Italian Societies of Neurology (SIN) and of Clinical Biochemistry and Clinical Molecular Biology - Laboratory Medicine(SIBioC) and from the Autonomous Association affiliated with SIN for dementia (SINdem)

**DOI:** 10.1007/s10072-026-08931-7

**Published:** 2026-03-21

**Authors:** Lorenzo Gaetani, Luisa Agnello, Andrea Pilotto, Alberto Benussi, Annachiara Cagnin, Lucilla Parnetti, Marco Bozzali, Alessandro Padovani, Marcello Ciaccio, the Italian Society of Neurology (SIN) Study Group on Biomarkers in Neurology, Marco Canevelli, Giordano Cecchetti, Giuseppe Di Fede, Giulia Giacomucci, Guido Maria Giuffrè, Piergiorgio Grillo, Daniele Imperiale, Camillo Marra, Alessandro Martorana, Federico Massa, Benedetta Nacmias, Federico Paolini Paoletti, Piero Parchi, Tommaso Piccoli, Domenico Plantone, Elisa Rubino, Martina Valletta, Gianluigi Zanusso, Laura Bonanni, the Italian Society of Clinical Biochemistry and Clinical Molecular Biology –Laboratory Medicine (SIBioC) Study Group on Neurological Markers, Giulia Musso, Giulia Sancesario

**Affiliations:** 1 Section of Neurology, Laboratory of Clinical Neurochemistry, University Hospital S. Maria Della Misericordia, Perugia, Italy; 2 Department of Biomedicine, Neurosciences and Advanced Diagnostics, Institute of Clinical Biochemistry, Clinical Molecular Medicine, and Clinical Laboratory Medicine, University of Palermo, Palermo, Italy; 3 Department of Clinical and Experimental Sciences, University of Brescia, Brescia, Italy; 4 Department of Continuity of Care and Frailty, Unit of Neurology, ASST Spedali Civili, Brescia, Italy; 5 Laboratory of Digital Neurology and Biosensors, University of Brescia, Brescia, Italy; 6 Neuromultibio Biorepository, University of Brescia and ASST Spedali Civili of Brescia, Brescia, Italy; 7 Center for Alzheimer Research, Division of Clinical Geriatrics, Department of Neurobiology, Care Sciences and Society (NVS), Karolinska Institut, Stockholm, Sweden; 8 Neurology Unit, Department of Medical, Surgical and Health Sciences, University of Trieste, 34149 Trieste, Italy; 9 Neurology Unit, Department of Medicine, Surgery and Health Sciences, Azienda Sanitaria Universitaria Giuliano Isontina, Trieste, Italy; 10 Department of Neuroscience (DNS) and Padua Neuroscience Centre (PNC), University of Padova, Padua, Italy; 11 Rita Levi Montalcini’ Department of Neuroscience, University of Torino, Turin, Italy; 12 SC Neurologia 2U, AOU City of Health and Science, Turin, Italy; 13 Department of Laboratory Medicine, University Hospital Paolo Giaccone, Palermo, Italy

**Keywords:** Alzheimer’s disease, Plasma biomarkers, P-tau217, P-tau217/Aβ42, Diagnostic framework, Clinical implementation, Italy

## Abstract

**Background:**

Blood-based biomarkers are reshaping the diagnostic paradigm of Alzheimer’s disease (AD), offering a minimally invasive alternative to cerebrospinal fluid (CSF) and positron emission tomography (PET). However, their clinical implementation requires harmonized analytical validation, standardized procedures, and clear guidance on appropriate use and interpretation.

**Methods:**

This joint position paper was developed by the Study Groups on Biomarkers in Neurology of the Italian Society of Neurology (SIN) and the Italian Society of Clinical Biochemistry and Clinical Molecular Biology – Laboratory Medicine (SIBioC), together with the Autonomous Association affiliated with SIN for Dementia (SINdem). A multidisciplinary panel of neurologists and laboratory experts systematically reviewed current evidence, international guidelines, and regulatory frameworks to formulate consensus recommendations for clinical adoption in Italy.

**Results:**

Among available plasma biomarkers, phosphorylated tau at threonine 217 (p-tau217), alone or as a ratio with amyloid-β42, shows the highest diagnostic accuracy, approaching CSF and PET performance. Fully automated platforms ensure analytical robustness and reproducibility. The document provides detailed guidance on pre-analytical handling, test selection, and reporting standards, emphasizing appropriate clinical indications and the need for contextual interpretation considering confounders such as advanced age or renal dysfunction.

**Conclusions:**

This position paper defines a unified framework for integrating plasma biomarkers into AD diagnostic pathways, bridging neurology and laboratory medicine. It highlights key steps toward regulatory inclusion in the Italian healthcare system and quality-assured clinical practice.

## Introduction: the context of Alzheimer’s disease diagnosis

Alzheimer’s disease (AD) is the most common cause of dementia globally, representing approximately 60–80% of all cases and posing a growing public health challenge as the world’s population ages [1]. Epidemiological projections estimate that the prevalence of AD will more than double by 2050, driven largely by demographic shifts and increased longevity, especially in Europe [2, 3]. Despite its high frequency, AD is not the only cause of progressive cognitive impairment: several other neurodegenerative conditions, as well as non-neurodegenerative disorders, can underlie similar clinical presentations. Relying on clinical assessment alone to identify AD is challenging [4, 5]. While episodic memory impairment is a hallmark feature, it is not specific to AD and may occur in other conditions [6]. Moreover, atypical clinical presentations of AD are not uncommon, further complicating the diagnostic process [7]. Pathologically, AD is defined by the extracellular accumulation of amyloid-β (Aβ) plaques and the intracellular aggregation of hyperphosphorylated tau into neurofibrillary tangles [8, 9]. These neuropathological changes emerge many years, often decades, before clinical symptoms become apparent, leading to a long preclinical phase characterized by progressive synaptic dysfunction, neuronal loss, and brain atrophy as tracked by a variety of biomarkers [8]. During the last two decades, both cerebrospinal fluid (CSF) and brain positron emission tomography (PET) biomarkers have transformed the diagnostic landscape by enabling in vivo confirmation of AD pathology even in prodromal phases [10, 11]. Core CSF biomarkers, Aβ peptide 1–42 (Aβ42), and its ratio with Aβ peptide 1–40 (Aβ40) (Aβ42/40 ratio), phosphorylated tau at threonine 181 (p-tau181) and total tau fragments (t-tau), are well validated, with high sensitivity and specificity in distinguishing AD from non-AD dementias [12–14]. Brain amyloid PET provides a non-invasive visualization of cortical amyloid deposition and is equally recognized as a gold-standard diagnostic tool [13]. In Europe and Italy, specific consensus guidelines have been developed for the biological definition of subjects with cognitive impairment based on structural brain imaging, PET imaging and CSF as main biomarkers included [13, 15]. The recent approval of disease-modifying anti-amyloid antibodies, such as lecanemab [16] and donanemab [17], both of which require biomarker confirmation of amyloid positivity before treatment initiation, has heightened the clinical importance of accurate, more accessible biomarkers [18, 19].

In this evolving landscape, both the Alzheimer’s Association (AA) 2024 criteria [10], and the International Working Group (IWG) 2024 recommendations [20], have formally recognized the role of plasma biomarkers as part of the diagnostic process for AD. The AA framework incorporates blood-based markers within the updated ATN (Amyloidosis, Tauopathy, Neurodegeneration) classification, acknowledging their utility especially in settings where CSF or PET are not readily available [10]. The IWG approach, while maintaining a clinical-biological definition of AD, also endorses the use of plasma biomarkers in support of a diagnostic hypothesis [20].

## Scope of the position paper

The Study Groups on Biomarkers in Neurology of the Italian Society of Neurology (SIN) and of the Italian Society of Clinical Biochemistry and Clinical Molecular Biology – Laboratory Medicine (SIBioC), together with the Autonomous Association affiliated with SIN for Dementia (SINdem), have jointly developed this position paper with the aim of providing practical recommendations and guidance on the use of plasma biomarkers for the diagnosis of AD, specifically tailored to the organization, regulatory framework, and clinical pathways of the Italian healthcare system. Rather than serving as a comprehensive review of the literature, this document is intended to represent a shared intersocietal reference to support harmonized clinical implementation and to inform organizational and public health perspectives at a national level.

Recent progress in the analytical performance and availability of these tests will be first summarized. The document will then address who should be tested, defining the clinical scenarios in which blood biomarkers are most appropriate, and who should request them, identifying the healthcare professionals and settings where their use is most beneficial. The biomarkers currently offering the best diagnostic performance will be reviewed, together with criteria for selecting the most suitable ones for each clinical context.

Because the accuracy of any laboratory test depends on the entire analytical workflow, procedures for sample handling and pre-analytical management will be discussed, as well as the analytical platforms that ensure robust and reproducible results. Finally, the interpretation of test results will be examined, emphasizing the importance of integrating laboratory data with clinical assessment in the post-analytical phase, and strategies for effectively communicating results to clinicians, individuals undergoing testing, and their caregivers will be outlined.

## Development of the position paper and consensus process

This position paper was developed through a structured inter-society process involving SIN, SINdem, and SIBioC. A core multidisciplinary working group of plasma markers and cognitive expert was selected by the Executive Committee of each Society (SIN/SINdem and SIBioC). The core multidisciplinary group initiated the project, reviewed the available literature, and prepared a first draft of the manuscript.

The draft was subsequently submitted to the Presidencies of the three societies, which independently coordinated an internal review process. This involved designated internal reviewers from each society (one for SIN, two for SINdem, and two for SIBioC), who provided scientific and methodological feedback. Following revision, the manuscript was shared with members of the Study Groups on Biomarkers in Neurology of SIN and SIBioC to allow further comments and suggestions.

Consensus was achieved through this multistep inter-society review and revision process. No formal Delphi procedure or voting-based consensus methodology was applied, as the primary objective of the document was to provide pragmatic, evidence-based guidance tailored to the Italian healthcare system rather than graded recommendations.

## Advances in plasma biomarkers for Alzheimer’s disease

Over the past decade, technological advances such as mass spectrometry, single-molecule array (Simoa), chemiluminescence (CLEIA) and electrochemiluminescence (ECLIA) immunoassays have enabled the quantification of AD-related proteins at the very low concentrations present in blood [21], thus opening the possibility of transferring diagnostic biomarkers from CSF to blood.

**Amyloid-β.** Mass spectrometry-based assays of plasma Aβ42/40 were the first to demonstrate strong correlations with amyloid PET and CSF Aβ42/40, with area under the curve (AUC) values ranging from 0.70 to 0.85 depending on platform and cohort [12, 22, 23]. While promising, performance has been more modest than that of CSF testing, partly due to peripheral metabolism of Aβ and the small relative differences (i.e. dynamic range) in plasma concentrations between amyloid-positive and amyloid-negative individuals [18]. As a result, despite significant group-level differences, the overlap between AD and non-AD individuals is substantial, making plasma Aβ markers alone insufficient for diagnostic purposes.

**Phosphorylated tau (p-tau).** In parallel, plasma phosphorylated tau species emerged as important biomarkers, with multiple studies showing high accuracy of p-tau181 in distinguishing AD from other neurodegenerative diseases and strong associations with amyloid and tau PET and CSF AD biomarkers [24, 25]. p-tau181 levels in plasma rise early in the disease course, track with disease progression, and correlate with established CSF and imaging markers, with an accuracy ranging from 0.75 to 0.90 for AD diagnosis compared to controls, depending on cohorts and techniques adopted [26–28]. Of note, the use of p-tau181/Aβ42 ratio appeared to slightly increase the performance compared to p-tau181 alone [18, 26]. Although p-tau181 offers robust performance, subsequent studies demonstrated that plasma p-tau217 outperforms p-tau181 in predicting amyloid PET status and in differentiating AD from other pathologies [29, 30]. Plasma p-tau217 has indeed consistently shown AUC values of 0.92–0.98 with different techniques and antibodies for identifying amyloid positivity, a performance comparable to CSF Aβ42/40 and p-tau181 [29, 31, 32]. Moreover, p-tau217 demonstrates a larger fold-change between amyloid-positive and amyloid-negative individuals than either plasma Aβ42/40 or p-tau181, making it more robust to analytical and biological variability [18]. Interestingly, combining p-tau217 with Aβ42 into a ratio, as tested for p-tau181, reduced the percentage of subjects with intermediate results [1], potentially via a normalization mechanism analogous to the Aβ42/40 ratio, providing a more accurate measure across individuals and disease stages.

Both plasma p-tau181 and p-tau217 are best interpreted as indicators of tau phosphorylation and secretion downstream of amyloid accumulation (T1), rather than as direct markers of tauopathy (T2), consistent with the most recent AA guidelines and recent biomarker staging frameworks [10]. Table 1 reports the diagnostic performance of plasma Aβ42/40, p-tau181, p-tau181/Aβ42, p-tau217, and p-tau217/Aβ42 using a single cut-off and using two reference values (two cut-offs strategy) maximizing sensitivity and specificity. Recent large-scale validation studies adopting a two cut-off t approach (low- and high-probability thresholds) have shown comparable performance, and in some cases even superior accuracy, exceeding 97.5% for p-tau217 [1].

**Table 1:** Overview of diagnostic performances in published studies including AD patients with CSF/amyloid PET confirmation using the two different approaches

Plasma biomarkers	Diagnostic performance (AUC)	References
	Single cut-off approach	
Aβ42/40	0.70–0.85	[12, 22, 25]
p-tau181	0.75–0.94	[24–29, 33, 34]
p-tau181/Aβ42	0.78–0.96	[26–28]
p-tau217	0.85–0.95	[1, 29, 31, 35]
p-tau217/Aβ42	0.88–0.98	[1]
	Two cut-offs approach (95%sensitivity and 95% specificity)	
p-tau217	0.89–0.94 Subjects with Intermediate levels: 12–29%	[1, 36]
p-tau217/Aβ42	0.86–0.92 Subjects with Intermediate levels: 8–10%	[1]

AUC: area under the curve. Aβ42/40: amyloid-β42/amyloid-β40. p-tau181: tau phosphorylated at threonine 181. p-tau217: tau phosphorylated at threonine 217. PET: positron emission tomography

### Other promising blood biomarkers

Additional candidates such as plasma tau phosphorylated at threonine 231 (p-tau231), microtubule-binding region tau 243 (MTBR-tau243), glial fibrillary acidic protein (GFAP), and neurofilament light chain (NfL) offer complementary information on tau phosphorylation, tauopathy, astrocytic activation, and neuroaxonal injury, respectively [26, 37, 38]. These markers have been proposed as potentially confirmatory or supportive tools in research settings with possible applications in disease staging, progression monitoring, or assessment of co-pathologies [39]. However, their implementation in clinical practice is currently limited either by the lack of widely scalable assays or by the insufficient availability of clinical evidence needed to define a clear context of use and robust interpretation framework.

## Clinical indications: who should be tested?

The current consensus is that plasma biomarkers should be used in individuals with objective cognitive impairment, either mild cognitive impairment (MCI) or dementia, when AD is among the possible underlying causes [40, 41]. This recommendation is grounded in evidence that plasma assays can meaningfully inform the diagnostic process when applied to the appropriate population and interpreted in the context of a comprehensive clinical evaluation. For subjects complaining of subjective cognitive decline or cognitively healthy individuals, evidence is conflicting and the clinical indication remains questionable, in line with current guidelines and recommmendations [10, 20, 40, 41]. As outlined in the international guidelines [13, 15], a specialist evaluation should be performed before requesting specific AD biomarkers. This evaluation should include at least a neurological assessment, comorbidities and frailty stratification, structural imaging and routine blood testing.

## Clinical settings: who should request the test?

Blood tests could be used in two distinct clinical settings: (i) as triage tools in primary care, intended here as specialist outpatient services in the community (e.g., neurology or geriatrics clinics within local health authorities), and (ii) as confirmatory tests in secondary care, referring to highly specialized hospital-based memory clinics, when performance thresholds of ≥ 90% sensitivity and specificity are achieved [40, 41].

Clinical sensitivity refers to the ability of the test to correctly identify individuals who truly have the disease (true positives). A highly sensitive test minimizes false negatives. Clinical specificity refers to the ability of the test to correctly identify individuals who do not have the disease (true negatives). A highly specific test minimizes false positives [42].

The diagnostic value of any biomarker, including blood-based tests, depends on its clinical sensitivity and specificity, but most importantly on its positive predictive value (PPV) and negative predictive value (NPV). PPV is the probability that an individual with a positive test result truly suffers from the disease. Negative predictive value (NPV) is the probability that an individual with a negative test result truly does not suffer from the disease [43]. While clinical sensitivity and specificity are intrinsic properties of the test (and to this regard, plasma p-tau217 alone or in the ratio with Aβ42 has shown sensitivity and specificity above 90%) [1, 44, 45], PPV and NPV depend on the pre-test probability (i.e., prevalence of the disease) [41]. In the case of AD, this probability is shaped by a combination of demographic variables (such as age) and the depth of the clinical evaluation preceding the test [1, 44, 46]. In clinical practice, this means that the same test result may have different implications depending on the setting and the level of suspicion for AD.

Multicenter studies explicitly contrasting primary/first-level and secondary/second-level cohorts show that identical plasma p‑tau–based tests (p‑tau217 alone or combined with Aβ42) yield very high accuracy in both settings, but the clinical actionability differs with pre-test probability [1]. In secondary care (higher suspicion), PPV reached 95%–96% and NPV 90–92%; in primary care/first-level cohorts, PPV was lower (83%–90%) and NPV 92%–96%, illustrating reliance of PPV/NPV on pre-test probability and the need for clinical context before establishing a biological diagnosis based on a positive result. PPV is lower in individuals with subjective cognitive decline and at early disease stages (lower prevalence), whereas NPV is higher, making the test more useful for ruling out AD pathology when clinical suspicion is modest, supporting redirection toward alternative etiologies [1].

Therefore, a positive plasma p-tau result, where the clinical suspicion may still be low or non-specific, is not sufficient on its own to establish a diagnosis of AD. Conversely, the same result, obtained where a thorough clinical workup has already raised a high suspicion of AD, may be confirmatory of a biological diagnosis. (Table 2).

**Table 2:** Clinical indications and interpretation of plasma biomarkers for AD in different care settings

	First-level setting*	Second-level setting**
Type of patient	Individuals with clinical suspicion of AD (any clinical stage)	Individuals with clinical suspicion of AD (any clinical stage)
Pre-test probability of AD	Low to moderate	Moderate to high
Use of plasma biomarkers	Triage tool to support/refute suspicion	Potentially sufficient to support biological diagnosis
Recommended biomarkers	p-tau217 or p-tau217/Aβ42	p-tau217 or p-tau217/Aβ42
Positive result	Suggestive but not conclusive of AD → requires further evaluation	Supportive of AD diagnosis if consistent with clinical and cognitive profile
Inconclusive results	Requires further evaluation	Requires further evaluation
Negative result	Useful to exclude AD pathology and consider alternative causes	Useful to exclude AD pathology and consider alternative causes
Interpretation caveats	Consider possibility of false positives due to comorbidities (e.g., CKD)	Consider test reliability; confirm in unclear cases
Analytical requirements	Prefer fully automated, CE-IVDR approved assays	Prefer fully automated, CE-IVDR approved assays
Clinical factors to assess	Age > 80, kidney function, differential diagnosis	Age > 80, kidney function, differential diagnosis, and possible access to confirmatory tests (CSF/PET)

**Legend:** *Centres where standard neuropsychological evaluation, routine blood tests, and structural neuroimaging can be performed or required and evaluated. **Specialised cognitive disorder centres where an advanced neuropsychological evaluation can be performed, with full access to CSF and brain PET biomarkers

Aβ42: amyloid-β peptide 1–42. AD: Alzheimer’s disease. CDCD: Center for Cognitive Disturbance and Dementia. CE-IVDR: *Conformité Européenne* – In Vitro Diagnostic Regulation. CKD: chronic kidney disease. CSF: cerebrospinal fluid. p-tau217: tau protein fragments phosphorylated at threonine 217. PET: positron emission tomography

Although plasma, CSF, and PET imaging biomarkers can, in principle, be used interchangeably for the biological diagnosis of AD, the choice of which test is more appropriate should be guided by clinical context, resource availability, and individual patient characteristics. CSF and amyloid PET imaging are established and broadly comparable for Aβ status, with plasma tests now enabling scalable front-end triage and, in some cases, confirmation depending on thresholds and setting [47, 48]. The AA authors similarly outline pathways that use blood tests to streamline access to disease-modifying treatments (DMTs) while reserving CSF/PET when needed [49].

For patients who are candidates for anti-amyloid therapies, an amyloid PET scan at baseline may be especially informative, both for confirming amyloid pathology and for establishing a biological reference point to evaluate treatment response/discontinuation over time. In this context, a diagnostic pathway that begins with plasma biomarkers and proceeds to amyloid PET assessment could fulfill both diagnostic and therapeutic planning needs [1, 48, 50]. The evolving scenario of current development of DMTs targeting amyloid-requiring biomarkers-based definition needs a cautious and pragmatic re-discussion of current national and international guidelines for AD diagnostic processes. The present position paper targets clinical and analytic definitions and context of use for blood-based AD biomarkers, not discussing in detail their specific time and role in the AD diagnostic algorithms, as this is specifically addressed by the Italian Intersociety working group on AD [19].

For the purposes of this paper, it should be noted that patient-specific factors may guide the preferred biomarker modality. These include medical conditions (e.g., coagulopathies, severe claustrophobia, anatomical barriers to lumbar puncture), comorbidities that may interfere with plasma biomarker accuracy (e.g., chronic kidney disease), and patient preferences related to invasiveness or exposure to radioactive tracers [51, 52]. Table 3 summarizes how individual factors may influence the choice between plasma, CSF, and PET biomarkers, as adapted for the Italian clinical context from VandeVrede et al. (2025) [51].

**Table 3:** Patient-specific factors guiding the choice of AD biomarkers (adapted from VandeVrede et al., 2025)

Patient-specific factors	Plasma biomarkers	CSF biomarkers	Amyloid PET
Patient is very concerned about risks from radiation	✓	✓	✗
Patient has severe claustrophobia	✓	✓	✗
Patient is treated with anticoagulant medications	✓	✗	✓
Patient is very concerned about invasiveness or risks of lumbar puncture	✓	✗	✓
Patient has risk factors for a difficult lumbar puncture (e.g., scoliosis, prior lumbar surgery, obesity)	✓	✗	✓
Differential diagnosis includes non-AD conditions better evaluated with CSF (e.g. motor neuron disease signs, subacute cognitive impairment)	✗	✓	✗
Patient is a candidate for anti-amyloid therapy requiring baseline documentation of amyloid pathology	~	~	✓
Only low-accuracy or poorly validated blood tests are accessible	✗	✓	✓
Patient has chronic kidney disease or other systemic and neurological comorbidities	✗	✓	✓

**Legend:** ✓ = Preferred option; ~ = Acceptable option; ✗ = Less suitable option

AD: Alzheimer’s disease. CSF: cerebrospinal fluid. PET: positron emission tomography

## Blood-based biomarkers for Alzheimer’s disease diagnosis: which test should be used?

As established by international guidelines, blood-based biomarkers with a sensitivity and specificity higher than 90% for AD diagnosis could be used for triaging or confirming AD [41].

The shift from research settings to fully automated platform with diagnostic value and diagnostic approval is dramatically changing the scenario of available markers in the market. Under the European regulatory framework, only assays with “*Conformité Européenne* – In Vitro Diagnostic” (CE‑IVD) marking, in compliance with the In Vitro Diagnostic Regulation (IVDR) EU 2017/746, can be used in clinical practice, making availability of certified kits a key criterion for adoption [53–55]. Assays should preferably be run on fully automated platforms to ensure reproducibility and scalability, as demonstrated by prospective, predefined‑cutoff studies using automated platforms with stable performance across primary and secondary care and biweekly runs [1, 40].

From a scientific standpoint, only plasma p-tau181 and p-tau217, either alone or in combination with their ratio to Aβ42, have achieved the minimum accuracy thresholds recommended by international guidelines for implementation as AD biomarkers [41]. To date, only two assays have received formal regulatory recognition from the United States (US) Food and Drug Administration (FDA). The “Elecsys Amyloid Plasma Panel” (Roche Diagnostics), which quantifies plasma p-tau181 together with apolipoprotein E4 (APOE4) [56], has been granted FDA Breakthrough Device Designation, whereas the “Lumipulse G pTau217/β-Amyloid 1–42 Plasma Ratio” (Fujirebio), which measures plasma p-tau217 and its ratio to Aβ42 [57], has received FDA 510(k) clearance (Table 4). According to European regulations, these assays are currently under evaluation for the CE-IVDR certification for their diagnostic use.

**Table 4:** Characteristics of the analytical platforms currently available for measuring plasma AD biomarkers

Assay	Platform	Manufacturer	Biomarkers	Regulatory status*	Pros	Cons
**CLEIA**	Lumipulse G System	Fujirebio	Aβ40, Aβ42, p‑tau181, p‑tau217, total tau	FDA 510(k) clearance (2025) for plasma p‑tau217/Aβ42 ratio assay (aid‑in‑diagnosis)	- High analytical sensitivity; Fully automated standardized workflow; High‑throughput random‑access testing	Requires dedicated Lumipulse instrumentation; Platform‑specific reagent cartridges
**Simoa**	HD‑X Analyzer	Quanterix	Aβ40, Aβ42, p‑tau181, p‑tau217	RUO	Ultra‑high analytical sensitivity; Very low sample volume; Widely used in research settings	High instrument and reagent cost; Requires experienced personnel; Dedicated instrumentation
	SP‑X planar multiplex platform	Quanterix	Aβ peptides, tau species	RUO	Multiplex capability suitable for exploratory biomarker panels	
**ECLIA**	Meso Scale Discovery (MSD)	Meso Scale Diagnostics	Aβ40, Aβ42, p‑tau181, p‑tau217, total tau	RUO	Multiplex capability; High analytical sensitivity; Customizable assay panels	Inter‑assay variability reported across laboratories; Greater assay optimization needs
	Cobas automated platform	Roche Diagnostics	Aβ40, Aβ42, p-tau181, total tau	CE‑IVD under IVDR (2025) for plasma p‑tau181 assay to support clinical rule‑out of AD FDA clearance (2025) for aid‑in‑exclusion of amyloid pathology	High analytical sensitivity; Standardized automated workflow; High‑throughput; widely implemented clinical platform	Requires dedicated cobas instrumentation
			p-tau217	RUO in process for FDA clearance		
	EUROIMMUN Analyzer I/EUROLabWorkstation	EUROIMMUN	Aβ40, Aβ42, p‑tau181, total tau	RUO	Fully automated processing; high analytical reproducibility; suitable for routine batch testing	Higher cost relative to manual ELISA systems; Platform‑specific reagents

ECLIA, Electrochemiluminescence Immunoassay; CLEIA, Chemiluminescent Enzyme Immunoassay; RUO, research use only; CE‑IVD, Conformité Européenne in vitro diagnostic certification; IVDR, In Vitro Diagnostic Regulation; AD, Alzheimer’s disease. Regulatory status refers to plasma‑based Alzheimer’s biomarker testing. The table summarizes analytical and regulatory characteristics of currently available platforms and does not imply comparative clinical superiority or cost-effectiveness among assays

## Pre-analytical considerations: how should samples be collected and handled?

The pre-analytical phase is a critical component of the total testing process in laboratory medicine, encompassing all procedures before sample analysis, including patient preparation, specimen collection, handling, transport, and storage. Despite often being overlooked, the pre-analytical phase is widely recognized as the most error-prone stage of laboratory testing, being responsible for up to 70% of all laboratory-related errors [58]. Its impact on the accuracy, reproducibility, and clinical interpretation of laboratory results is profound. Thus, efforts have been made to define and standardize pre-analytic variables for blood-based AD biomarkers [59, 60].

Starting from the patient preparation for blood collection, the impact of individual-level pre-analytical variables, such as fasting status, physical activity, medication use, and time of day of blood collection, on the plasma AD biomarkers has been poorly explored. Some authors have shown that plasma p-tau217, Aβ40, and Aβ42 levels vary with time of day, with the lowest levels observed in the morning upon waking and the highest values in the afternoon/early evening for p-tau217 [61]. Additionally, food intake may influence the plasma biomarkers levels, with significant differences observed between fasting and postprandial states [62]. However, no definite conclusion can be drawn due to the few data available. The impact of blood draw devices has not yet been explored [63]. However, to standardize blood collection procedures across laboratories, O’Bryant et al. recommend the use of 21-gauge needles for venipuncture in adults [64].

Blood AD biomarkers should be measured in plasma because their levels are decreased in serum due to loss from clot trapping, which occurs during the coagulation process required to generate serum [65]. This phenomenon is particularly pronounced for Aβ, which are known to adhere to the fibrin clot, resulting in artificially lower concentrations in serum compared to plasma, leading to underestimation of true circulating biomarker levels. The choice of anticoagulant is another important aspect influencing the plasma biomarker levels. Among all, EDTA is the anticoagulant of choice, while lithium heparin and sodium citrate are not recommended because they are associated with altered levels of plasma AD biomarkers [66].

Time from blood collection to centrifugation, particularly under room temperature or cold conditions, represents the main source of pre-analytical variation. Delays in processing whole blood samples, especially beyond 6–24 h, lead to a marked decrease in Aβ40 and Aβ42 concentrations, with reductions observed as early as 4 h at room temperature and after 24 h at 4 °C. The Aβ42/40 ratio can partly compensate for this effect but can still be affected by prolonged delays, potentially leading to misclassification or reduced assay sensitivity for amyloid pathology.

For plasma phosphorylated tau (p-tau181 and p-tau217), levels are more stable than Aβ, but p-tau181 can increase with pre-centrifugation delays of 24 h or more, especially at room temperature, which may result in falsely elevated values. To ensure accuracy and reliability of plasma measurements for these biomarkers, centrifugation should ideally occur within 2 h of collection, and samples should be kept at 4 °C if immediate processing is not possible. Delays beyond this window, especially at room temperature, compromise the stability of Aβ and tau. Optimal centrifugation is also essential for high-quality plasma preparation, as inappropriate speed or duration can lead to cell lysis or incomplete separation of plasma from blood cells. Most studies report centrifuging for 5–15 min at 1500–3000 × g and room temperature. Thus, Verberk et al. recommend centrifuging at 1800× g for 10 min at room temperature [67].

The timing of analysis is another critical factor in ensuring the reliability of AD plasma-based biomarkers. Both short- and long-term storage conditions can significantly impact biomarker stability, and thus, influence the accuracy of measurement. When immediate analysis is not possible, plasma samples can be stored at 4 °C for up to 24 h without significant degradation. However, it is strongly recommended to minimize the time between centrifugation and freezing, as post-centrifugation delays, even at 4 °C, can result in a gradual decline in analyte levels. To mitigate this, samples should be kept on wet ice during short-term holding periods and frozen as soon as possible. Plasma should be analysed within 24 h if stored at 4 °C or up to two weeks if stored at −20 °C [67]. For prolonged storage prior to analysis, plasma samples should be aliquoted into low-protein-binding tubes with O-ring seals and stored at − 80 °C or in liquid nitrogen tanks, as long-term storage at − 20 °C is associated with increased risk of protein degradation and evaporation over time. Studies have shown that AD biomarkers remain stable for several months to years under ultra-low temperature conditions if proper protocols are followed.

**Fig. 1:**
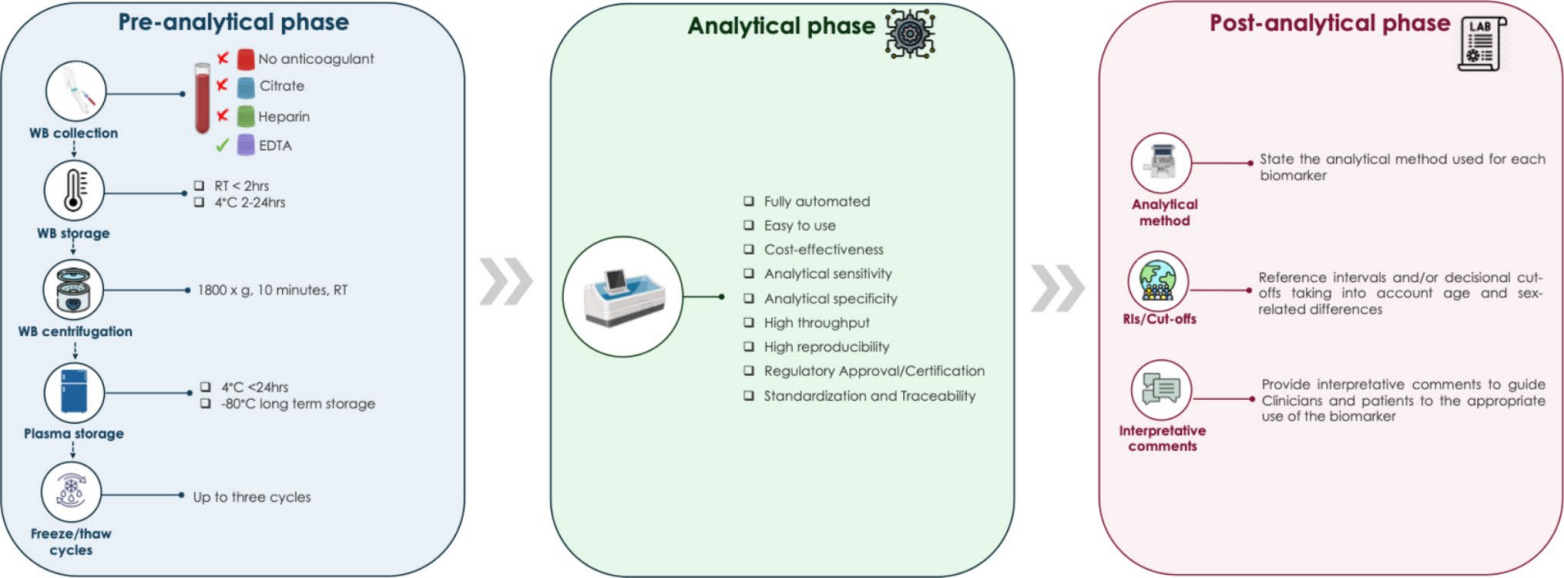
Laboratory testing process of plasma biomarkers of Alzheimer’s disease. WB, whole blood; RIs, reference intervals. The process spans the pre-analytical, analytical, and post-analytical phases, including whole-blood collection preferring the plasma, centrifugation (1800 × g, 10 min), and plasma storage (≤ 24 h at 4 °C or long-term at − 80 °C; up to three freeze–thaw cycles); analytical measurement using automated, high-throughput and standardized platforms with validated sensitivity, specificity, and reproducibility; and post-analytical reporting that should specify the analytical method, reference intervals and/or clinical cut-offs (accounting for age and sex), and interpretative comments to guide clinical use of the biomarkers

Delays following centrifugation can also lead to reduced biomarker levels, though the rate of decline is generally slower compared to delays before centrifugation. Finally, several authors explored the impact of freeze/thaw cycles on blood-based biomarkers, showing that plasma levels are stable up to three freeze/thaw cycles [67]. Figure 1 describes the laboratory testing process for plasma AD biomarkers, from pre-analytical to analytical and post-analytical phase.

## Analytical phase: how should assays and platforms be chosen and validated?

Analytical methods for measuring AD plasma biomarkers have evolved rapidly over the past two decades. The turning point in the field came with the development of digital immunoassay technologies, most notably the Simoa platform in 2010. This technology represented a breakthrough by enabling the detection of proteins at femtomolar concentrations. By utilizing arrays of femtoliter-sized reaction chambers, the Simoa platform significantly improves assay sensitivity and specificity, while also reducing matrix interference. This innovation allowed for more reliable quantification of plasma Aβ42, and p-tau181, establishing a new benchmark for blood-based biomarker analysis in AD.

The introduction of immunoprecipitation coupled with mass spectrometry (IP-MS) marked another milestone in the field. It combines the selectivity of antibody capture with the molecular specificity of liquid chromatography–tandem mass spectrometry (LC–MS/MS), providing high precision for detecting AD-related peptides, including Aβ isoforms and phosphorylated tau variants, in plasma samples. IP-MS has demonstrated excellent diagnostic accuracy for autopsy-confirmed AD, with plasma p-tau217 showing the highest performance for distinguishing AD from non-AD cases and correlating with neuropathological staging [68]. It also enables detection of disease-relevant changes at preclinical and prodromal stages, supporting early diagnosis and trial recruitment [69]. However, its technical complexity, high cost, and limited scalability compared to immunoassays hampers its implementation in clinical practice.

To bridge the gap between analytical precision and clinical scalability, fully automated immunoassay platforms have emerged as a practical solution. Among these, electrochemiluminescence immunoassays (ECLIA) such as the Elecsys system (Roche Diagnostics, Penzberg, Germany), the EUROIMMUN® ECL immunoassay (EUROIMMUN, Luebeck, Germany), and the Mesoscale Discovery (MSD) platform (Meso Scale Diagnostics, Rockville, MD, USA) have demonstrated performance comparable to digital immunoassays for the measurement of plasma Aβ and p-tau species, while offering the advantages of high throughput, reproducibility, and ease of integration into routine laboratory workflows.

Another critical contributor is the Lumipulse G System (FUJIREBIO Inc., Tokyo, Japan), a fully automated chemiluminescent enzyme immunoassay (CLEIA) platform. Initially validated and approved for the analysis of CSF biomarkers, the Lumipulse platform has recently been adapted for plasma-based detection of AD biomarkers. Its automated workflow, robust performance, and increasing clinical validation make Lumipulse one of the most viable solutions for routine diagnostic use [70] (Table 4). Key features of analytical methods for clinical implementation include sufficient analytical sensitivity to accurately detect pathophysiologically relevant concentrations, even at preclinical disease stages; high reproducibility in terms of low intra- and inter-assay variability, which are essential to ensure that diagnostic thresholds and cut-offs are reliable, regardless of geographic or institutional setting; automation, which is essential to minimize human error, ensure consistent pre-analytical and analytical handling, and enable high-throughput testing; and regulatory approval and standardization.

Overall, the successful implementation of an analytical method in clinical practice requires more than technical excellence, demanding a balanced integration of scientific rigor, operational feasibility, and regulatory compliance. Platforms like Lumipulse (Fujirebio) and Elecsys (Roche) are leading examples that align closely with these criteria, marking a transition toward more accessible, reliable, and minimally invasive diagnostics for AD. Recent regulatory advances have led to the first clinically approved blood-based biomarkers for AD [71]. In May 2025, the U.S. Food and Drug Administration (FDA) granted 510(k) clearance to the “Lumipulse® G pTau217/β-amyloid 1–42 Plasma Ratio” assay (Fujirebio) as the first blood test intended to aid in the diagnosis of AD by identifying amyloid pathology in adults with cognitive impairment [72]. In Europe, the Elecsys® pTau181 plasma assay (Roche Diagnostics, in collaboration with Eli Lilly) received CE-IVD certification under the In Vitro Diagnostic Regulation (IVDR) on 23 July 2025 as the first IVDR-approved blood test to support the clinical “rule-out” of AD when used in conjunction with clinical assessment. Subsequent FDA clearance in 2025 further expanded its use for aiding exclusion of AD-related amyloid pathology in symptomatic patients. Together, these approvals mark a major step toward the integration of minimally invasive plasma biomarkers into routine diagnostic pathways, offering a more accessible alternative to PET and CSF testing.

Finally, a major challenge in the clinical implementation of plasma biomarkers lies in achieving proper standardization and traceability. Standardization refers to the harmonization of assay procedures, calibrators, and data interpretation, ensuring consistency across different laboratories and over time. Traceability, on the other hand, links measurement results to established reference materials or methods, allowing for reliable cross-platform comparisons. These aspects are particularly critical for AD biomarkers, where diagnostic thresholds may directly influence patient management. However, for key analytes such as Aβ42, Aβ40, and p-tau isoforms, the lack of certified reference materials and cross-platform variability remain significant obstacles. In addition, biological complexity, including the existence of multiple isoforms and post-translational modifications, as well as matrix effects in plasma (e.g., interference from high-abundance proteins), further complicates assay standardization. Although international initiatives such as the Global Biomarker Standardization Consortium (GBSC) and working groups under the International Federation of Clinical Chemistry (IFCC) have made important progress, full traceability and global standardization of plasma-based assays for AD remain unmet goals. Nonetheless, they are essential for widespread clinical adoption and for enabling meaningful comparisons across trials and real-world settings.

## Post-analytical interpretation: how should biomarker data be integrated into the clinical context?

The post-analytical phase of laboratory testing represents a critical step in ensuring the appropriate interpretation of laboratory data [73]. The ISO 15189:2023 standard for medical laboratory accreditation defines the post-analytical phase as the “processes following the examination, including review of results, formatting, releasing, reporting and retention of examination results, retention and storage of clinical material, the sample and waste disposal” [74]. Thus, a laboratory report is fundamental for converting raw numerical data into information helpful to clinicians in the decision-making process and patient management. To provide meaning and value to laboratory data, beyond the basic information related to patient attendance, the report should contain measurement units, analytical method used, reference intervals, and/or decisional cut-offs, and interpretative comments, when needed. Especially for clinical neurochemistry, which is a relatively young field of laboratory medicine, the comment is critical for guiding clinicians and patients to the appropriate interpretation. Indeed, many clinicians, also from high-income countries like the United Kingdom and Spain, lack confidence in using or interpreting these tests, indicating the need for careful planning if biomarker testing becomes more widely available [75].

### Cut-offs for plasma Alzheimer's disease biomarkers

Currently, there is no universally accepted reference standard for determining plasma biomarker cut points [76]. Studies often use different assay platforms, measurement units, and population samples, leading to different thresholds [77–79]. This heterogeneity prevents direct comparison between studies and hinders the development of unified diagnostic criteria. In addition, most plasma biomarker cut-offs are derived by correlating plasma levels with amyloid-PET results. However, PET imaging itself lacks uniform thresholds, with reported amyloid positivity values varying between 13.5 and 24 Centiloids across studies [80]. This variability introduces uncertainty in defining what constitutes a “true” positive or negative case, affecting how plasma cut points are interpreted and validated. In addition, several methods could be used to define cut points. A single cut-point approach offers simplicity and clear binary classification (positive/negative), often determined using statistical tools like the Youden Index. However, in real-world settings, biomarker distributions overlap between individuals with and without AD pathology. This overlap results in indeterminate results, reducing clinical confidence and often requiring further confirmatory testing with CSF or PET.

For the reasons described above, several international working groups have proposed the use of two cut-points to define three categories of results: positive, intermediate, and negative [1, 38]. This approach is currently implemented in the FDA-approved Lumipulse plasma p-tau217/β-Amyloid 1–42 assay [40]. Such an approach allows the same biomarker to be used both for triaging and confirmatory purposes by applying different cut-offs optimized for sensitivity (triaging) or specificity (confirmation) [40].

For FDA approval, the “Lumipulse G pTau 217/β-Amyloid 1–42 Plasma Ratio” assay defined the following interpretive cut-offs, based on a validation cohort of about 550 subjects: ≤ 0.00370 negative, indicating the patient is unlikely to have amyloid pathology; 0.00371 to 0.00737 indeterminate zone, meaning additional diagnostic evaluation is recommended; ≥ 0.00738 positive, suggesting the patient is likely to have amyloid pathology [40].

In Europe, a recent larger multicentre validation study in primary vs secondary settings calculated 95% sensitivity and specificity cut-offs based on a population of more than 1200 subjects with CSF/Amyloid PET confirmation [1]. For the two-cut-off approach, p-tau217/Aβ42 cut-offs were: the lower 95% sensitivity cut-off < 0.007 (95% CI 0.006–0.008) and the upper 95% specificity cut-off > 0.009 (0.008–0.011). For p-tau217 alone, the work also calculated a lower cut-off at < 0.22 (95% CI 0.17–0.25) and an upper cut-off at > 0.34 (95% CI 0.30–0.41) pg/mL yielding the same accuracy but higher percentage of intermediates [1]. Of note, these cut-offs were independent from age, sex, kidney function (excluding severe cases) and vascular comorbidities [1]. These real-life based cut-offs might reduce the number of intermediates compared with the one submitted to the FDA but need to be verified in ongoing international validation studies.

For other platforms/biomarker with high accuracy but lack of available cut-offs, each center should establish its own cut-offs based on the selected reference outcome (e.g., amyloid PET, CSF A +, or CSF A + T +), ideally with a higher and lower cut-off maximizing sensitivity and specificity, respectively. Where this is not feasible, reference can be made to cut-offs generated in large, multicenter cohorts with similar populations, or to those submitted to regulatory agencies by the manufacturers (Table 4).

### Clinical considerations for test interpretation

When interpreting plasma biomarker results, the clinician should consider several factors that may affect the test’s reliability and accuracy. The most important ones are age, renal function and differential diagnosis, while no definite evidence is available on the influence of sex and body mass index (BMI) [76–78].

### Age-related decline in diagnostic accuracy after ~ 80 years

High prevalence of amyloid positivity in cognitively normal older adults, affecting over 40% of individuals aged 80–89, is well documented. In such cases, amyloid detection may indicate a risk factor for cognitive decline rather than an inevitable path to dementia [80]. This weakens the positive predictive value of plasma AD biomarkers in this population and introduces clinical ambiguity, especially in asymptomatic individuals. Community-based and clinic cohorts show increasing overlap of plasma p‑tau181 and p‑tau217 between AD and non-AD at advanced ages, with attenuated discrimination relative to younger-old groups; accuracy remains useful but is lower in the oldest-old, warranting cautious interpretation and a lower threshold for confirmatory testing when results are borderline or discordant with phenotype. Neuropathology-verified and PET-anchored studies consistently find p‑tau217 superior to p‑tau181 overall, but emphasize validation in older, unselected populations due to this overlap [30, 33, 39, 45, 81]. Beyond chronological age, emerging evidence from CSF and PET AD biomarker studies suggests that measures of biological aging, such as frailty, may provide additional information for interpreting biomarker test results and for patient stratification [82, 83]. However, these findings still need to be confirmed for blood-based AD biomarkers.

### Renal function as a confounder

Renal dysfunction (higher creatinine/reduced eGFR) raises plasma p‑tau181 and, to a lesser extent, p‑tau217 independent of brain amyloid/tau, increasing false-positive risk. Effects are most evident in CKD stage ≥ 3 and in Aβ − individuals. Two complementary approaches mitigate this: (a) use p‑tau217-to-unphosphorylated tau ratios (pT217/T217), which markedly attenuate CKD associations while preserving AD signal; this approach requires at the moment mass spectrometry measures; and (b) consider composite tests, such as ratios, that are less sensitive to CKD. Adjusting formulas for severe CKD are under investigation, as mild kidney alterations are known not to consistently affect values and cut-offs [1, 17, 84]. In the presence of CKD or borderline/uncertain results, confirmatory CSF or PET is recommended to avoid misclassification [34, 81, 85–87].

### Differential diagnosis and potential false positives

Elevated plasma p‑tau has been reported in other neurodegenerative conditions, such as amyotrophic lateral sclerosis, likely reflecting peripheral/motor system sources (e.g., amyotrophic denervation), which can reduce specificity for AD in such populations [88–90]. Rapidly progressive dementias (e.g., prion diseases) can also show increased plasma p‑tau species [91], further underscoring the need to interpret results in clinical context and to use confirmatory biomarkers when phenotype is atypical or progression is rapid. Head‑to‑head assay work indicates that low–molecular‑weight, brain-enriched p‑tau assays improve specificity versus assays that detect peripheral high–molecular‑weight tau, which is relevant when peripheral neuromuscular disease is present [88–90, 92–94]. However, at present these biomarkers are not ready for routine clinical use.

**Fig. 2:**
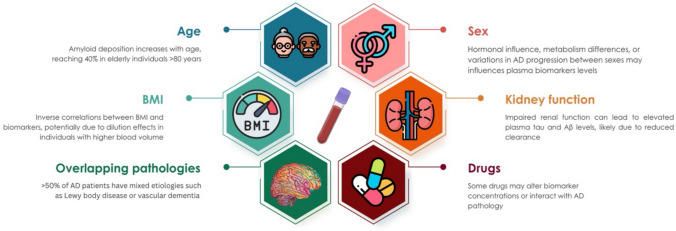
Biological and clinical factors influencing plasma biomarkers of Alzheimer’s disease (AD). Several individual characteristics and comorbid conditions may affect plasma biomarker concentrations and their interpretation. Age is associated with increasing amyloid deposition, reaching high prevalence in individuals over 80 years. Sex-related hormonal and metabolic differences, as well as variation in disease progression, may influence biomarker levels. Higher body mass index (BMI) shows inverse associations with biomarker concentrations, potentially due to dilution effects in individuals with greater blood volume. Impaired kidney function can lead to elevated plasma tau and Aβ levels through reduced clearance. More than half of patients with AD present overlapping pathologies, such as Lewy body disease or vascular dementia, which may modify biomarker profiles. In addition, some medications may alter biomarker concentrations or interact with AD pathology

Biological variability, coexisting conditions, and age-related changes all impact test accuracy (Fig. 2). To prevent misdiagnosis, overdiagnosis, or patient confusion, a multidisciplinary model must remain central in biomarker-based dementia care.

### Laboratory report drafting, interpretation of findings with patients and caregivers

The formulation of final reporting should include the individual biomarker levels, the cut-offs used, and a general interpretation statement for the results and the platform/technique used. The panel discussed and proposed the following statement based on different diagnostic categories:

a) positive result: “the biomarkers are indicative of amyloid pathology”.

b) negative result: “the biomarkers are not indicative of amyloid pathology”.

c) intermediate result: “the biomarkers are not conclusive for confirming or excluding amyloid pathology and warrants further assessment”.

A repetition in blood AD markers is not formally indicated in intermediate cases if no concerns about the preanalytical/analytical phases are present [17]. Despite several studies showed a mild to moderate increases of AD markers levels during disease course, the repetition of the blood AD testing is not indicated, as the short/middle term fold change of biomarkers is low [17], and the subtle biomarkers changes did not show any relevance for the clinical management [33].

The final report findings/results of the assessment should be discussed by a clinician expert of cognitive impairment (ideally, the same team who prescribed the blood-based biomarkers) and the individual, together with the caregiver when possible. In accordance with current biomarkers guidelines [13, 14], the findings should be reported in the appropriate setting and with sufficient time to discuss their impact on pharmacological and non-pharmacological approaches and implications for general management and care.

**Table:** 

BOX – Panel Recommendation for blood AD testing in Italy
Who is the prescriber of a blood AD test?
A clinician, specialist of cognitive disorder after clinical assessment of the patient
Which clinical requirements are needed prior to testing?
The individual should undergo neurological examination, cognitive assessment, comorbidity and frailty screening, structural imaging and routine blood testing before testing
Which is the target population for AD blood testing?
Subjects with objective cognitive deficits (either mild cognitive impairment or dementia) Subjects symptomatic for cognitive disturbances for whom the clinician consider clinically useful the use of fluid biomarkers
Which biomarkers should be used?
Currently, p-tau217/Aβ42 ratio and p-tau217 alone reached the highest accuracy
For diagnostic purposes, CE-IVD tests should be preferred to RUO tests
**Which key factors should be considered as potentially affecting biomarker levels?**
Severe kidney disfunction
Age older than 80 years
How should blood sampling be performed?
The use of 21-gauge needles for venipuncture in adults is recommended
Plasma is the matrix of choice
EDTA is the anticoagulant of choice
Centrifugation should occur within 2 h of collection; samples should be kept at 4 °C if processing is not possible
Centrifugation should be performed for 5–15 min at 1500–3000 × g and room temperature
Plasma should be analysed within 24 h if stored at 4 °C or up to two weeks if stored at −20 °C
How should the findings be reported?
The reporting should include the date of test, the biomarker levels, the cut-offs used, and the platform/technique used
The reporting should be accompanied by a general interpretation of the findings
Who should explain and discuss the results of findings?
A clinical specialist in cognitive disorders
In which settings should be presented/discussed the biomarkers?
The findings should be reported in the appropriate setting and with sufficient time to discuss their impact for diagnostic/therapeutic strategies
Is the repetition of biomarkers indicated during the course of the disease?
Currently, there is no indication for repetition of biomarkers during the follow-up. In case of baseline indeterminate results, however, a second test 6 to 12 months later could be considered

Aβ42: amyloid-β peptide 1–42. AD: Alzheimer’s disease. CE-IVD: *Conformité Européenne*. In Vitro Diagnostic. p-tau217: tau protein phosphorylated at threonine 217. RUO, Research Use Only

## Current challenges and future perspectives

In this position paper, we have outlined the current evidence on plasma biomarkers for AD, detailing their analytical performance, pre-analytical requirements, and optimal clinical contexts for use within the European healthcare setting, specifically in Italy. The panel has identified p-tau217, alone or in a ratio with Aβ42, as the most accurate and ready-to-implement blood-based markers, recommended in carefully defined patient populations and preferably using CE-IVD–certified assays. We have also set out clear procedural, reporting, and interpretive standards, summarized in the final recommendations box, to ensure consistency and quality across all stages of testing, from prescription to clinical discussion of results.

From a regulatory perspective, it will first be necessary to obtain the CE-IVD mark [54]. Subsequently, the Italian National Outpatient Service Tariff List (*Nomenclatore Tariffario Nazionale delle prestazioni di specialistica ambulatoriale)* will need to be updated so that plasma biomarkers for the diagnosis of AD are included in the Essential Levels of Healthcare (*Livelli Essenziali di Assistenza*—*LEA*) and can be reimbursed within the Italian National Health Service. This is an essential and non-deferrable update, and we hope to raise awareness among the relevant national authorities to prioritize its implementation.

Once regulatory and reimbursement frameworks are in place, it will be crucial to design an accreditation system for laboratories performing these tests. Accredited centers should participate in national and international quality control programs, ensuring standardization and reproducibility of results [39, 47, 95–97]. In this initial phase, a centralized approach might be preferred, with testing limited to laboratories already experienced in CSF biomarker diagnostics for AD, as this will guarantee an adequate level of expertise and quality assurance [96].

While current consensus focuses on patients with objective cognitive impairment, emerging evidence supports the careful, ethically grounded exploration of biomarker testing in asymptomatic individuals at elevated risk, such as double *APOE* ε4 carriers, individuals with type 2 diabetes or insulin resistance [98], or those with a strong family history or genetic conditions [99], within appropriately designed clinical frameworks. Such an extension will be justifiable if the literature confirms a clear predictive value of these biomarkers, and if testing remains guided by specialist evaluation. At this stage, their application to large-scale screening programs appears unrealistic, at least until effective, codified, widely applicable, and economically sustainable DMTs are available for clinical use.

While this paper has primarily addressed the diagnostic value of plasma biomarkers, future research may expand their utility to prognostic assessment and monitoring of treatment response. Currently, robust prognostic data are lacking for the biomarkers considered here, although plasma markers of axonal, glial, or synaptic damage have proven promising in this direction [26, 100–102]. Similarly, early-phase data suggest that certain plasma biomarkers, such as p-tau217, decrease in individuals treated with anti-amyloid antibodies [50]. However, given that these biomarkers do not reflect the cumulative pathology present in the central nervous system as PET imaging does, it is premature to recommend their use for monitoring therapeutic response [50, 103]. Ongoing research into novel plasma assays may change this scenario in the coming years.

The consensus will remain open to revisions and implementations, aligning with fast development and validation of new platforms with high standards of accuracy and diagnostic validity. A special interest will be the development of next-generation multiplex technologies, enabling simultaneous measurements of multiple molecular targets including AD-specific pathology [104]. These advances will inevitably prompt a re-examination of existing diagnostic algorithms, with the aim of integrating multi-marker strategies with the ability of capturing the complexity of mixed and atypical pathologies at single subject level. To ensure that such innovations translate into reliable clinical decision-making algorithms, national implementation has to be accompanied by standardized quality-control programs, harmonized pre-analytical and analytical procedures, and development of certified reference materials to secure cross-platform and cross-setting comparability [13, 14]. Embedding these measures within a coordinated national framework will not only uphold scientific and analytical rigor but also facilitate sustainable, patient-centered care pathways in the era of precision diagnostics.

## Data Availability

Not applicable.

## Abbreviations

Aβ: Amyloid-β
Aβ40: Amyloid-β peptide 1–40
Aβ42: Amyloid-β peptide 1–42
Aβ42/40: Ratio of amyloid-β 1–42 to amyloid-β 1–40
AA: Alzheimer’s Association
AD: Alzheimer’s disease
ADNI: Alzheimer’s Disease Neuroimaging Initiative
APOE/APOE4/APOE ε4: Apolipoprotein E / ε4 allele
AUC: Area under the receiver operating characteristic curve
BMI: Body mass index
CDCD: *Centro Disturbi Cognitivi e Demenze*
CE: *Conformité Européenne*
CE-IVD: *Conformité Européenne* – In Vitro Diagnostic
CE-IVDR: *Conformité Européenne* – In Vitro Diagnostic Regulation (EU 2017/746)
CKD: Chronic kidney disease
CLEIA: Chemiluminescent enzyme immunoassay
CSF: Cerebrospinal fluid
DMT: Disease-modifying therapy
ECLIA: Electrochemiluminescence immunoassay
EDTA: Ethylenediaminetetraacetic acid
eGFR: Estimated glomerular filtration rate
EU: European Union
FDA: U.S. Food and Drug Administration
GFAP: Glial fibrillary acidic protein
IFCC: International Federation of Clinical Chemistry
IP-MS: Immunoprecipitation mass spectrometry
ISO: International Organization for Standardization
IWG: International Working Group
LEA: *Livelli Essenziali di Assistenza*
MCI: Mild cognitive impairment
MSD: Meso Scale Discovery
MTBR-tau243: Microtubule-binding region tau 243
NfL: Neurofilament light chain
NPV: Negative predictive value
NVS: Neurobiology, Care Sciences and Society (Karolinska Institutet)
PET: Positron emission tomography
PPV: Positive predictive value
p-tau181: Tau phosphorylated at threonine 181
p-tau217: Tau phosphorylated at threonine 217
p-tau231: Tau phosphorylated at threonine 231
p-tau181/Aβ42: Ratio of p-tau181 to Aβ42
p-tau217/Aβ42: Ratio of p-tau217 to Aβ42
RUO: Research Use Only
SABB: Standardization of Alzheimer’s Blood Biomarkers
SIBioC: Italian Society of Clinical Biochemistry
Simoa: Single molecule array
SIN: Italian Society of Neurology
SINdem: Italian Dementia Association (autonomous association affiliated with SIN)
SP-X: Simoa SP-X assay system
t-tau: Total tau
T217/pT217/T217: Total tau 217 and phosphorylated-to-total tau 217 ratio
WB: Whole blood
